# The Combination Treatment of Curcumin and Probucol Protects Chondrocytes from TNF-*α* Induced Inflammation by Enhancing Autophagy and Reducing Apoptosis via the PI3K-Akt-mTOR Pathway

**DOI:** 10.1155/2021/5558066

**Published:** 2021-06-24

**Authors:** Guangtao Han, Yubiao Zhang, Haohuan Li

**Affiliations:** Department of Orthopedics, Renmin Hospital of Wuhan University, Wuhan, Hubei 430060, China

## Abstract

Osteoarthritis (OA) is a chronic joint disease characterized by cholesterol accumulation in chondrocytes, cartilage degeneration, as well as extracellular matrix (ECM) destruction, and joint dysfunction. Curcumin, a chemical that can reduce cholesterol levels in OA patients, also can inhibit the progression of OA. However, a high concentration of curcumin may also trigger apoptosis in normal chondrocytes. Besides curcumin, probucol that is found can also effectively decrease the cholesterol level in OA patients. Considering that high cholesterol is a risk factor of OA, it is speculated that the combination treatment of curcumin and probucol may be effective in the prevention of OA. To investigate the possible effects of such two chemicals on OA pathophysiology, chondrocyte apoptosis and autophagy behavior under inflammatory cytokine stress were studied, and specifically, the PI3K-Akt-mTOR signaling pathway was studied. *Methods*. Cell proliferation, colony formation, and EdU assay were performed to identify the cytotoxicity of curcumin and probucol on chondrocytes. Transwell assay was conducted to evaluate chondrocyte migration under TNF-*α* inflammation stress. Immunofluorescence, JC-1, flow cytometry, RT-PCR, and western blot were used to investigate the signal variations related to autophagy and apoptosis in chondrocytes and cartilage. A histological study was carried out on OA cartilage. Glycosaminoglycan (GAG) release was determined to evaluate the ECM degradation under stress. *Results*. Compared with a single intervention with curcumin or probucol, a combined treatment of these two chemicals is more effective in terms of protecting chondrocytes from stress injury induced by inflammatory cytokines. The promoted protection may be attributed to the inhibition of apoptosis and the blockage of the autophagy-related PI3K/Akt/mTOR pathway. Such results were also verified in vitro by immunofluorescence staining of OA chondrocytes and in vivo by immunohistochemistry staining of cartilage. Besides, in vivo studies also showed that when applied in combination, curcumin and probucol could block the PI3K-AKT-mTOR signaling pathway; promote COL-II expression; suppress P62, MMP-3, and MMP-13 expression; and inhibit TNF-*α*-stimulated cartilage degradation. Moreover, the combined medication could help reduce the release of ECM GAGs in OA cartilage and alleviate the severity of OA. *Conclusion*. A combined treatment of curcumin and probucol could be used to protect chondrocytes from inflammatory cytokine stress via inhibition of the autophagy-related PI3K/Akt/mTOR pathway both in vitro and in vivo, which might be of potential pharmaceutical value for OA prevention and therapy.

## 1. Introduction

OA is a chronic inflammatory disease closely related to cartilage degeneration. Researchers have found that a high level of total cholesterol is related to the OA process. Specifically, in a prospective cohort study, total cholesterol and triglycerides are verified to be associated with new bone marrow lesion formation in asymptomatic middle-aged women [1] and result in cartilage defect and OA eventually. Another possible explanation could be lipid embolism caused by serum cholesterol, which may cause osteonecrosis leading to OA.

Hypertension, obesity, abnormal blood lipids, and high cholesterol, such conditions known as “metabolic syndrome” [2] are common among OA patients. The interrelationship between high cholesterol levels and increased risk of OA has been studied extensively in recent years [3, 4], and previous reports have shown that inhibition of de novo cholesterol synthesis may provide better OA remiment outcome [5, 6]. In this context, OA should be considered as a syndrome rather than merely a joint disease.

Autophagy is an important self-maintenance mechanism by which a cell protects itself when facing harmful stress [7]. Active autophagy is related to cholesterol effluent, and it can delay disease progress to a certain alleviated extent. Specifically, nitro-oleic acid, a ligand of CD36, reduces cholesterol accumulation by modulating fluidized LDL uptake and cholesterol efflux in RAW264.7 macrophages, and FGF21 induces autophagy-mediated cholesterol efflux to inhibit atherogenesis via the upregulation of RACK1 [8].

However, autophagy activity tends to drop in several cells and tissues with age. In OA chondrocytes, autophagy markers decrease significantly [9], accompanying with dysfunctional autophagy, enhanced apoptosis, and less migration [10]. Therefore, chemicals that can regulate autophagy in chondrocytes and stabilize the cholesterol level may be of potential medication value for OA prevention and therapy.

Curcumin, a diferuloylmethane, is extracted from the root of *Curcuma longa* [11]. In China and India, *Curcuma longa* has been used as a medicinal herb with a long history. Recent research indicated that curcumin can function to reduce cholesterol levels [12]. Additionally, previous authors have verified that curcumin can promote autophagy and reduce apoptosis in several cells [13]. Moreover, probucol, another cholesterol regulator, can also activate autophagy and inhibit apoptosis in nerve cells by blocking PI3K/Akt/mTOR signals [14]. Since autophagy takes an important role in chondrocyte physiology, and the PI3K/Akt/mTOR pathway is essential in regulating autophagy in OA patients [15], we speculated that curcumin and probucol may be of potential value for OA prevention. In the present study, these two chemicals were applied together to investigate their effects on chondrocytes in vitro and on cartilage in vivo.

## 2. Materials and Methods

### 2.1. Animals

Healthy male Sprague Dawley rats from the Animal Experimental Center of Wuhan University (Wuhan, China) were involved in this study. The rats were fed under specific pathogen-free conditions at a constant room temperature (24°C) and relative humidity (45%–55%). All rats had free access to sterile food and water and lived under a light/dark cycle of 12 h. The present study was approved by the Laboratory Animal Welfare & Ethics Committee, Renmin Hospital of Wuhan University. Efforts were made to minimize animal suffering in the study.

### 2.2. Reagents

The reagents included DMEM/F12 high glucose (Hyclone, Utah, USA), penicillin (Hyclone, Utah, USA), streptomycin (Hyclone, Utah, USA), curcumin (Bellancom, Beijing, China), trypsin (Google Biotechnology, Wuhan, China), collagenase-II, bovine serum albumin (BSA), probucol (Sigma-Aldrich, St. Louis, MO, USA), KeyFluor488 Click-iT EdU kits, DAPI, (KeyGEN BioTECH, Nanjing, China), AnnexV-PI kits (BD, USA), Counting Kit-8 (CCK-8) reagents, goat serum (Beyotime Institute of Biotechnology, Shanghai, China), TNF-*α* (Peprotech, Inc., Suzhou, China), Caspase-3, Bcl-2, Lc3, Bax, PI3K, p-PI3K, Akt, p-Akt, mTOR, p-mTOR, GADPH, Berclin-1, COL-II, P62, FITC, Cy3, MMP-3, JC-1 assay kits (Abcam, USA), TRIzol reagents (Invitrogen, Thermo Fisher Scientific, Inc. USA), a RevertAid First Strand cDNA Synthesis kit (Fermentas; Thermo Fisher Scientific, Inc. USA), MMP detection kits (Solarbio Science & Technology, Beijing, China), and chemiluminescent luminol reagent (Santa Cruz Biotechnology, Texas, USA).

### 2.3. Chondrocyte Culture and Identification

Briefly, cartilage was extracted from the knee joints of 35 male Sprague-Dawley rats (4 weeks, weighing 140 ± 10 g). Cartilage samples were minced into thin slices (1 mm^3^) and digested with 3 ml of 0.25% trypsin for 40 min followed by further treatments with type II collagenase for another 6 h. Chondrocytes were then been centrifuged and collected. Subsequently, the isolated chondrocytes were cultured in 5 ml of DMEM/F12 with 20% fetal bovine serum and incubated at 37°C in 5% CO_2_.

### 2.4. CCK-8 Assay

To determine the appropriate study concentration of probucol and curcumin for further investigation in the subsequent experiments, cell viability was detected by the CCK-8 test. The chondrocytes were first cultured in a 96-well plate, and CCK-8 reagents were added, which was incubated at 37°C for another 2 h. The chondrocyte viability was detected by OD 450 nm with an automatic microplate reader. All studies were conducted in triplicate.

### 2.5. Cell Groups

Based on the above CCK-8 results, cells were randomly divided into five groups (*n* = 3): control, TNF-ɑ, TNF-ɑ + curcumin (50 *μ*M), TNF-ɑ + probucol (100 *μ*M), and TNF-ɑ + probucol (50 *μ*M) + curcumin (25 *μ*M). After excluding other cytokines or growth factors, TNF-*α* aqueous solution (20 ng/ml) was mixed with normal chondrocytes to mimic the inflammatory cytokine environment in OA [16]. 36 h later, curcumin, probucol, or both of them were added, and the chondrocytes were further incubated for another 24 h.

### 2.6. Flow Cytometry of Annexin V-FITC-PI Staining

The apoptosis rates of chondrocytes were measured with an AnnixV-PI apoptosis detection kit. In short, the chondrocytes were held at 25°C for 15 min and treated with PI solution (5 *μ*l) and FITC-labeled annexin V (5 *μ*l) for 10 min in the dark. The apoptosis rates were evaluated with a flow cytometer (BD Biosciences, USA).

### 2.7. Colony Formation Assay

The chondrocytes were placed on a six-well plate and mixed with curcumin and probucol at predescribed concentrations. After that, the cells were incubated for another two weeks without curcumin and probucol. Subsequently, the colonies were fixed with methanol, stained with Wright-Giemsa solution, and counted for their numbers [17].

### 2.8. Transwell Migration Assay

Transwell assays were used to evaluate cell migration. First, the transwell chambers were washed with serum-free medium, and chondrocytes were cultured in DMEM medium with 10% FBS as the chemical attractant. After incubation for 48 h, cells attached to the membrane were discarded, and those entering the lower membrane were fixed with methanol and stained with 0.2% crystal violet. Under a microscope (×200), the cells invaded by the matrix gel in 5 random fields of view were photographed.

### 2.9. JC-1 for Mitochondrial Membrane Permeability (MMP) Assessment

An MMP detection kit was used to evaluate the MMP in chondrocytes. After the chondrocytes were washed with PBS, 800 *μ*l of JC-1 working fluid was mixed with the chondrocytes and stained at 37°C for 25 minutes. Subsequently, 2 ml of medium containing serum was added to the working fluid after staining. The red-green fluorescence ratio was measured by a FACS Caliber flow cytometer (Becton, Dickinson, and Company) and an Olympus fluorescence microscope (Olympus Corporation, Japan).

### 2.10. EdU Incorporation Assay

Chondrocyte proliferation was assessed by a keyFluor488 Click-iT-EdU kit. First, the chondrocytes were placed in a six-well plate, and 100 *μ*l of EdU was added into the plate, followed by incubation at 37°C for 2 h. Second, the cells were fixed with 4% paraformaldehyde at room temperature, washed with BSA containing 3% glycine, and incubated with 0.5% TritonX-100 and 1× click-it reaction solution in the dark at room temperature. Last, Hoechst 33342 was added to the six-well plate, and the whole plate was placed in a dark environment for 20 minutes and then washed three times with PBS. The stained cells were observed with a fluorescence microscope.

### 2.11. Reverse Transcription Quantitative Polymerase Chain Reaction (RT-PCR)

TRIzol reagents were used to isolate the total RNA from chondrocytes. To determine the expression levels of inflammation-related genes, first-strand complementary cDNA chains were synthesized using the RevertAid First Strand cDNA Synthesis kit (Fermentas; Thermo Fisher Scientific, Inc.). Quantitative PCR was performed for 40 cycles in a StepOnePlus device (Applied Biosystems; Thermo Fisher Scientific, Inc.), and each cycle contained temperature at 95°C for 10 secs, followed by 5 seconds at 95°C and 20 seconds at 60°C. The additional primers were as follows: COL2, 5′-CTTAGGACAGAGAGAGAAGG-3′; Rev, 5′-ACTCTGGGTGGCAGAGTTTC-3′; MMP-3, 5′-TTTGGCCGTCTCTTCCATCC-3′; Rev, 5′-GGAGGCCCAGAGTGTGAATG-3′; MMP-13, 5′-GG AGCATGGCGACTTCTAC-3′; Rev, 5′-GAGTGCTCCAGGGTCCTT; GADPH, 5′-CTCAACTACATGGTCTACATGTTCCA-3′; and Rev, 5′-CTTCCCATTCTCAGCCTTGACT-3′. GADPH was used as an internal reference. Moreover, the 2-*ΔΔ*Cq method was employed to calculate the relative levels of mRNA expression.

### 2.12. Western Blot

To extract the total proteins from the chondrocytes, organophosphorus inhibitors, protease inhibitors, and RIPA lysates were mixed at a ratio of 1 : 1 : 50. The proteins were separated by electrophoresis and transferred to polyvinylidene fluoride membranes, which were sealed for one hour. After that, a primary antibody was added to the membranes, which were then washed three times with TBST and incubated with horseradish peroxidase-labeled anti-rabbit goat IgG for 1 hour. Subsequently, the membranes were washed with TBST again, and the protein bands were observed with chemiluminescent luminol reagent (Santa Cruz Biotechnology, Inc.) and an Image Lab quantitative analysis system (Bio-Rad Laboratories Inc.). The relative protein levels were compared by normalizing to GADPH. The primary antibodies were as follows: Bcl-2, Bax, Beclin-1, LC3, mTOR, PI3K, Akt, p-Akt, p-PI3K, p-mTOR, and GAPDH.

### 2.13. OA Animal Model In Vivo Study

SD rats (8 weeks old, weighing 250-280 g) were randomly divided into five groups, which are denoted as control (*n* = 12), OA (*n* = 12), OA+50 mg/kg curcumin (*n* = 12), OA+100 mg/kg probucol (*n* = 12), and OA+75 mg/kg curcumin-probucol (*n* = 12). The specific dosages were determined according to the earlier literature [18]. A rat OA model was created by excising the medial meniscus and the anterior cruciate ligament of the rats' right knee. Four weeks later, the groups with medications were treated with curcumin and probucol intramuscular injections once every three days for a total of 8 weeks, while the OA and the control groups were injected with normal saline. All rats were sacrificed after 3 months.

### 2.14. Immunofluorescence and Immunohistochemistry

After washed with PBS, the cartilage tissues and chondrocytes were fixed with paraformaldehyde for 12 h at 4°C and then dehydrated in 30% sucrose solution. Next, the tissues were sliced into pieces of 10 *μ*M and incubated with P62 and COL-II at room temperature for 1 h. Subsequently, the section slices were then immunostained with FITC or Cy3-labeled secondary antibodies for 1 h, and DAPI was applied to counterstain the nuclei for 5 min. The sections were then incubated overnight with the primary antibodies for MMP-3 or MMP-13 at 4°C, and they were then incubated with biotinylated secondary antibodies. All sections were observed under an Olympus fluorescence microscope mentioned above. The proportions of stain-positive cells in the samples were analyzed by Image Pro Plus 6.0 (Media Cybernetics, Inc., USA).

### 2.15. Glycosaminoglycan Release Assay

Papain-digested cartilage explants and their defrosted supernatants were examined in 96-well plates using the dimethyl methylene blue (DMMB) method [16]. Briefly, each sample was diluted in distilled water to a total volume of 40 *μ*l per well in triplicate. Shark chondroitin sulfate (Sigma-Aldrich) was used as a standard (0-70 ng). DMMB solution (200 *μ*l) was added to each well, and the whole plate was immediately transferred to a Multiskan Ascent Scanner (Thermo Labsystems, Basingstoke, UK) with Ascent Software (version 2.6, Thermo Labsystems, Finland). Total GAG release was observed from a spectrophotometric reading of the digested cartilage and its supernatants at 540 nm. For each well, the percentage of GAG release was calculated by dividing the GAG readings from the supernatants by the total GAG release.

### 2.16. Statistical Analysis

For each group, the data are expressed as means ± SD. Intragroup differences were assessed with Student's *t*-test and one-way analysis of variance by SPSS 16.0 (SPSS, Inc., USA) followed by a Bonferroni posthoc correction for multiple testing with GraphPad Prism (version 7.04; GraphPad Software, Inc., USA). Specifically, differences with *P* < 0.05 were considered statistically significant.

## 3. Results

### 3.1. Effects of Probucol and Curcumin on Chondrocyte Proliferation

**Figure 1:**
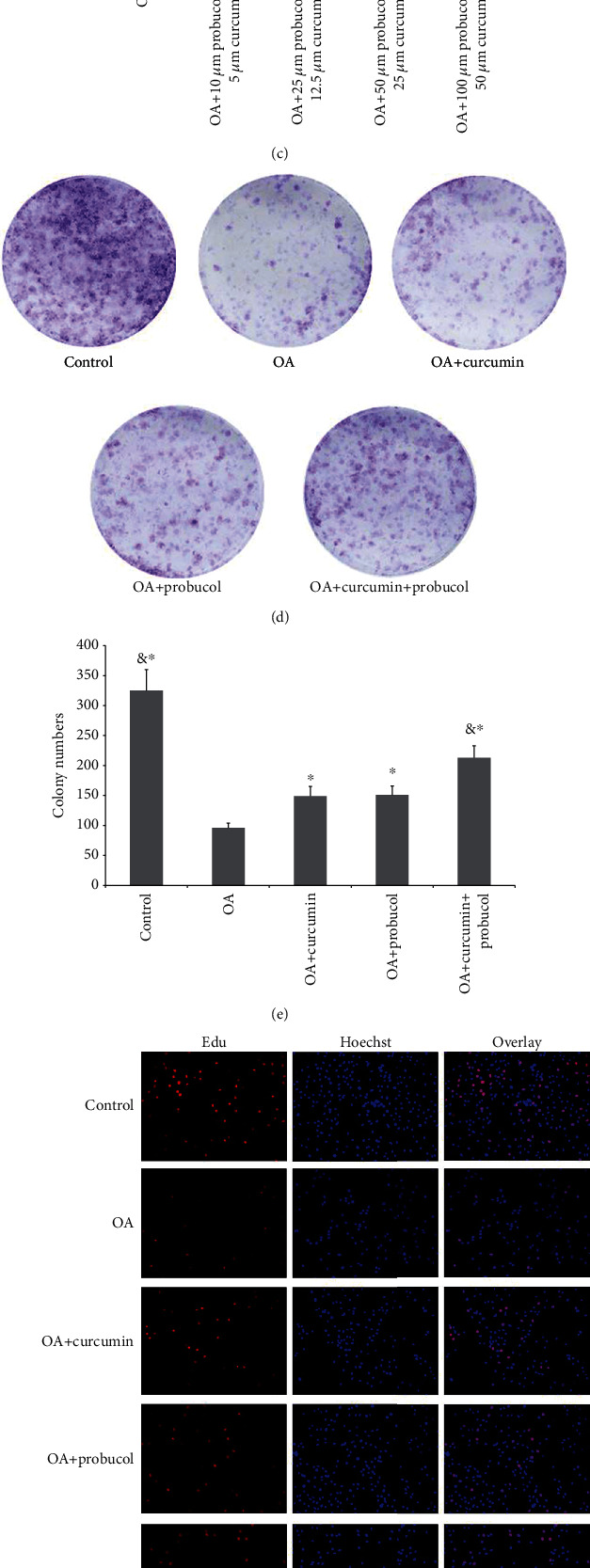
Probucol and curcumin enhance chondrocyte proliferation. (a) Cell viability at different probucol concentrations. (b) Cell viability at different curcumin concentrations. (c) Cell viability at different curcumin-probucol concentrations. (d) After incubated in 96-well plates, the cells were subsequently treated with curcumin and probucol. Cell proliferation in the control, OA, OA + curcumin, OA + probucol, and OA + curcumin + probucol groups was measured by colony formation assays. (e) Statistical analysis of the number of chondrocyte colonies. (f) Compared with the OA group, more EdU-labeled cells exhibited red fluorescence in the curcumin and probucol groups. Therefore, when applied together, curcumin and probucol would significantly reduce the number of EdU-labeled cells compared with either the curcumin or probucol groups. All cell nuclei displayed blue fluorescence with Hoechst33342 staining (magnification ×200). (g) The relative ratio of EdU-positive cells in the curcumin + probucol group was significantly higher than the other four groups at 24 h. The data are shown as the means ± SD. & and ∗ indicate *P* ≤ 0.01 and 0.05 vs. OA, respectively. All experiments were carried out three times.

CCK-8 was used to detect chondrocyte activity. The most appropriate concentrations of probucol and curcumin to counteract inflammatory cytokine stress were found to be 100 *μ*M and 50 *μ*M, respectively (Figures 1(a) and 1(b)). It is noteworthy that both these substances could promote chondrocyte proliferation in a dose-dependent manner. Here, we chose these substances at optimal concentrations of 12.5%, 25%, 50%, and 100% to the most appropriate concentration for combinations [10]. Considering the possible reported side effects of such substances [19], a combination of curcumin 25 *μ*M + probucol 50 *μ*M was used in this study, and the results suggest that such a combination can promote chondrocyte proliferation (Figures 1(c)). Colony formation assays further confirmed that they play a promotive role in chondrocyte proliferation (Figures 1(d) and 1(e)), and such effect is in a synergistic way by the two chemicals. In the EdU assays with TNF-*α* treatment, the chondrocytes showed a low proliferation ratio. However, after treating with 50 *μ*M curcumin or 100 *μ*M probucol, the proliferation ratio got increased; and with the combined treatment, such increasement became more significant (Figures 1(f) and 1(g)).

### 3.2. Effects of Probucol and Curcumin on Apoptosis and Apoptosis-Related Genes in Chondrocytes

**Figure 2:**
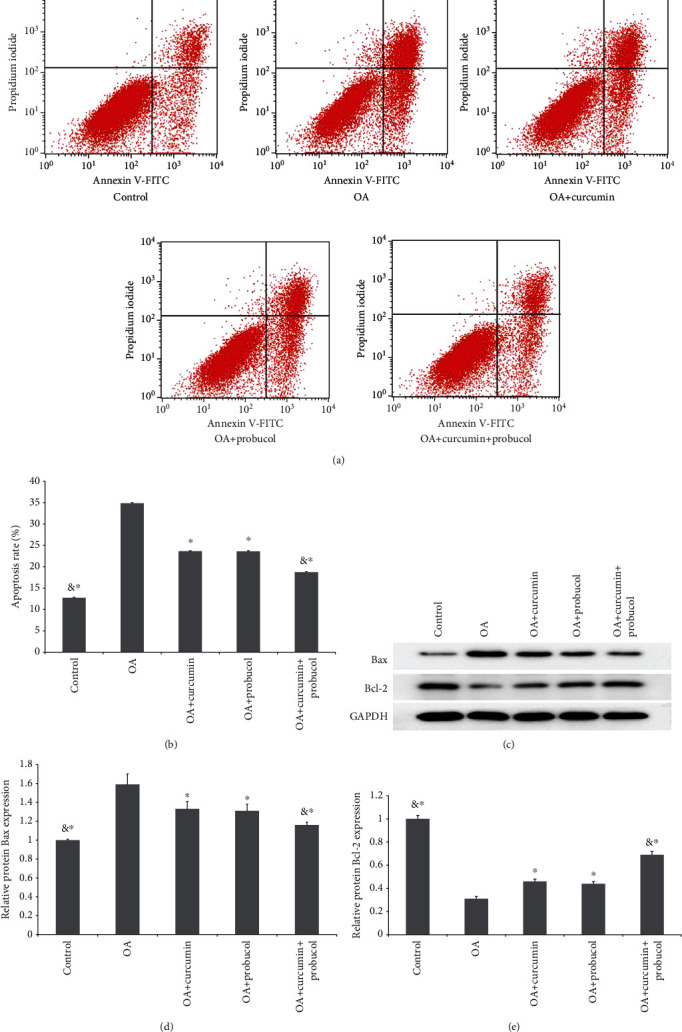
Probucol and curcumin can reduce chondrocyte apoptosis. (a) Probucol and curcumin inhibit apoptosis in chondrocytes. (b) The cell apoptosis rate significantly decreased after a treatment of curcumin + probucol for 24 h. (c) Bcl-2 and Bax protein concentration in chondrocytes (from Western blot assays). (d) Analysis of Bax expression based on the Western blot results. (e) Analysis of Bcl-2 expression based on the Western blot results. The data are shown as the means ± SD. & and ∗ indicate *P* ≤ 0.01 and 0.05 vs. OA, respectively. All experiments were carried out three times.

AnnexV/PI was used to detect chondrocyte apoptosis. It was found that TNF-*α* induced chondrocyte apoptosis (34.92% ± 0.75%). However, with 50 *μ*M curcumin or 100 *μ*M probucol addition, the TNF-*α*-induced apoptosis percentages dropped to 23.58% ± 0.6% and 23.49% ± 0.55%, respectively. Moreover, the apoptosis ratio of chondrocytes decreased significantly to 17.45% ± 0.45% in the curcumin + probucol group (Figures 2(a) and 2(b)).

Besides the apoptosis-inducing effects, TNF-*α* can also downregulate the expression of the Bax gene while upregulating Bcl-2 expression. Compared with the OA group, both the curcumin and probucol groups showed lower Bax expression, and the expression of Bcl-2 in these two groups was higher than that in the TNF-*α* group. Furthermore, the expression of apoptosis-related proteins was significantly lower in the curcumin + probucol group compared with the TNF-*α* group (Figures 2(c)–2(e)). All experiments were carried out three times.

### 3.3. Effects of Probucol and Curcumin on Chondrocyte Migration

**Figure 3:**
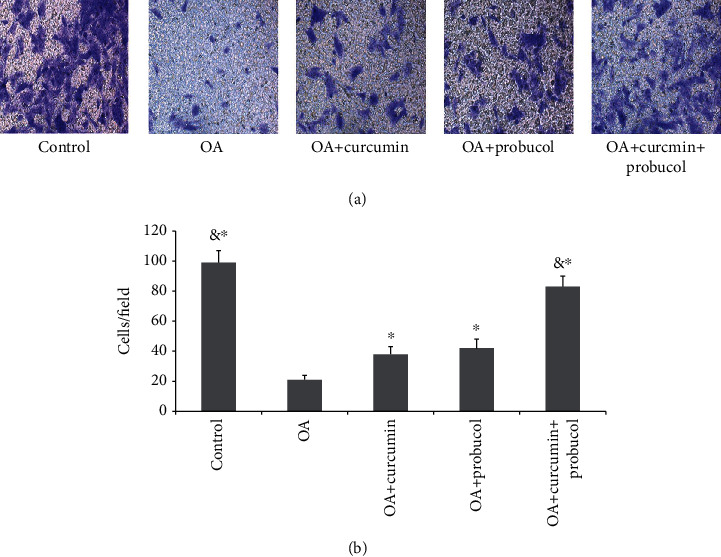
Probucol and curcumin enhance chondrocyte invasion. (a) Photographs of chondrocytes taken under a microscope (magnification ×200). (b) The migration rates were calculated based on the number of chondrocytes. The data are shown as means ± SD. & and ∗ indicate *P* ≤ 0.01 and 0.05 vs. OA, respectively. All experiments were carried out three times.

Transwell assays were used to observe cell migration, and TNF-*α* has been verified to reduce the mobility of chondrocytes. Compared with the TNF-*α* group, the curcumin and probucol groups were found to promote chondrocyte migration, and such promotive effect became more significant in the curcumin + probucol group. Eventually, chondrocyte mobility in the curcumin + probucol group was similar to that of normal chondrocytes (Figures 3(a) and 3(b)).

### 3.4. Effects of Probucol and Curcumin on the Mitochondria in Chondrocytes

**Figure 4:**
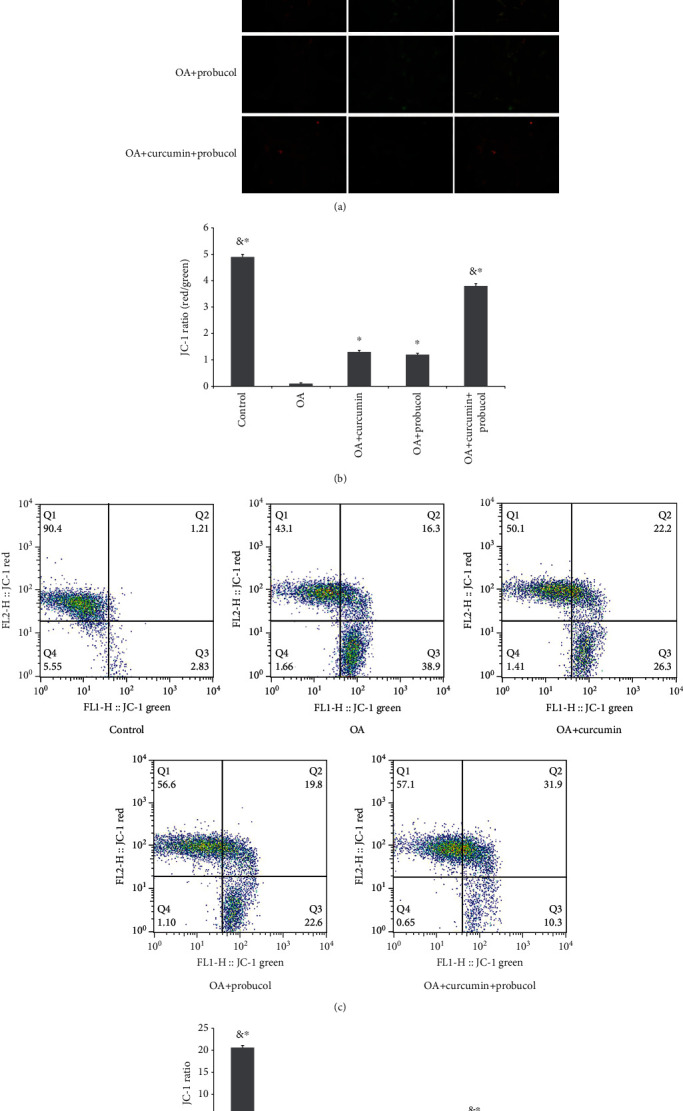
Probucol and curcumin increase the mitochondrial membrane potential of chondrocytes. (a) Changes in the chondrocyte MMP were observed by fluorescence microscopy (magnification ×200). Red fluorescence suggested normal MMP and green fluorescence indicated MMP decrease or loss. (b) The JC-1 ratios were calculated based on the number of chondrocytes. (c and d) Quantitative PNS analysis on *Δψ* m of chondrocytes. The data are shown as the means ± SD. & and ∗ indicate *P* ≤ 0.01 and 0.05 vs. OA, respectively. All experiments were carried out three times.

Mitochondria play an important role in energy production, cell signal transduction, cell differentiation, and apoptosis. The mitochondrial membrane potential (*Δψ* m) is an important parameter of mitochondrial function, which is used as an indicator of cell viability and usually changes when apoptosis occurs. The effects of probucol and curcumin on OA chondrocytes were influenced by apoptosis, which depends on mitochondria. JC-1 assays were performed to measure *Δψ* m. In a JC-1 assay, dyes would stay in the cytoplasm and emit green fluorescence in apoptotic cells; while in normal cells, dyes would gather in the mitochondria and emit red fluorescence. The results showed that the green fluorescence was brighter in the TNF-*α* group, suggesting that TNF-*α* can reduce *Δψ* m of chondrocytes and induce apoptosis. However, curcumin and probucol can increase *Δψ* m, and such increases in the two substances were found to be synergistic by flow cytometry (Figures 4(a)–4(d)). All experiments were carried out three times.

### 3.5. Effects of Probucol and Curcumin on the Activity of Autophagy-Related Proteins and the Autophagy-Related PI3K/Akt/mTOR Pathway

**Figure 5:**
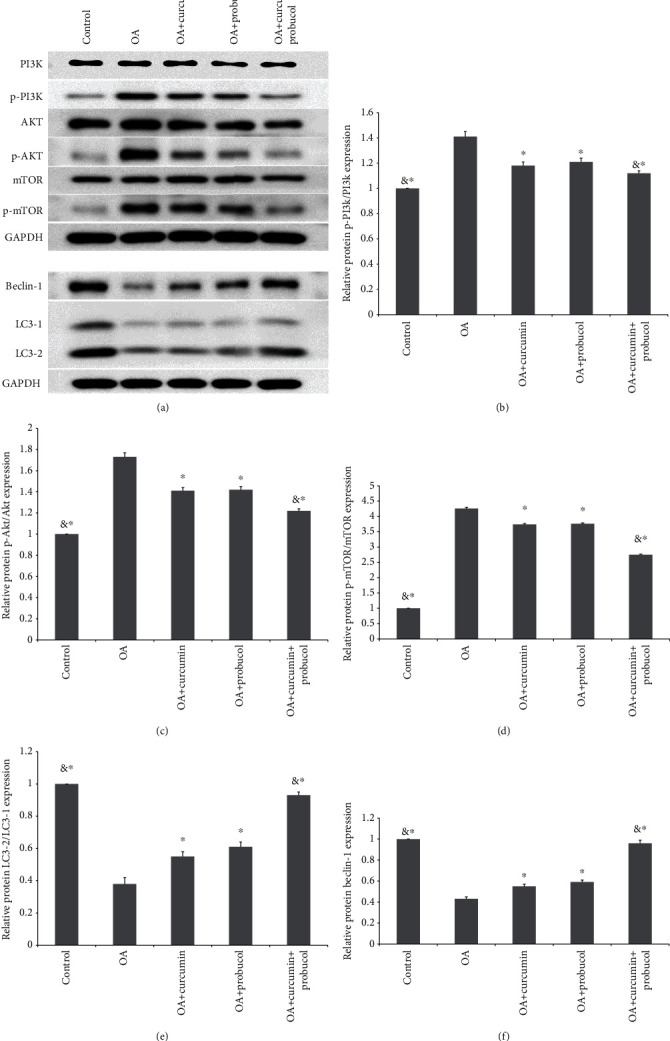
Probucol and curcumin inhibited OA by targeting the PI3K/Akt/mTOR pathway in chondrocytes. (a–f) Western blot results for quantitative analysis of PI3K, p-PI3K, Akt, p-Akt, mTOR, and p-mTOR protein expression in chondrocytes. The data are shown as the means ± SD. & and ∗ indicate *P* ≤ 0.01 and 0.05 vs. OA, respectively. All experiments were carried out three times.

**Figure 6:**
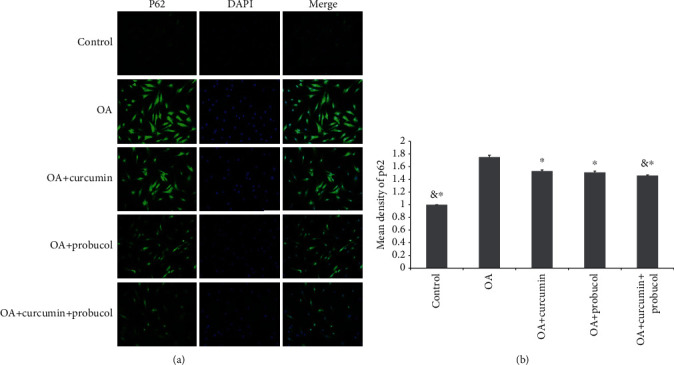
(a and b) Immunofluorescence staining revealed the amount of P62 protein in the chondrocytes. The data are shown as the means ± SD. & and ∗ indicate *P* ≤ 0.01 and 0.05 vs. OA, respectively. All experiments were carried out three times.

PI3K/Akt/mTOR is an important pathway in autophagy. To study the effects of curcumin and probucol on the expression of autophagy-related proteins, chondrocytes were cultured for 24 hours, then TNF-*α*, TNF-*α* +50 *μ*M curcumin, TNF-*α* +100 *μ*M probucol, and TNF-*α* +50 *μ*M probucol +25 *μ*M curcumin were mixed with the chondrocytes and incubated for 24 hours. Western blot assays were used to evaluate the expression of PI3K, p-PI3K, Akt, mTOR, p-Akt, p-mTOR, and autophagy-related proteins, such as LC3 and beclin-1. Besides, the autophagy-related factor P62 was detected by immunofluorescence. Compared with the OA group, probucol and curcumin could significantly reduce the phosphorylation degrees of p-PI3K, p-Akt, and p-mTOR, while significantly increasing the amount of autophagy-related proteins (Figures 5(a)–5(f)). Such effects became stronger when the two substances were applied together. Immunofluorescence showed that the expression of P62 decreased significantly. Therefore, the results suggested that probucol and curcumin regulate the mTOR signaling pathway through the PI3K-Akt pathway to promote autophagy and inhibit apoptosis of chondrocytes (Figures 6(a) and 6(b)).

### 3.6. Effects of Probucol and Curcumin on the Metabolism of Cartilage ECM

**Figure 7:**
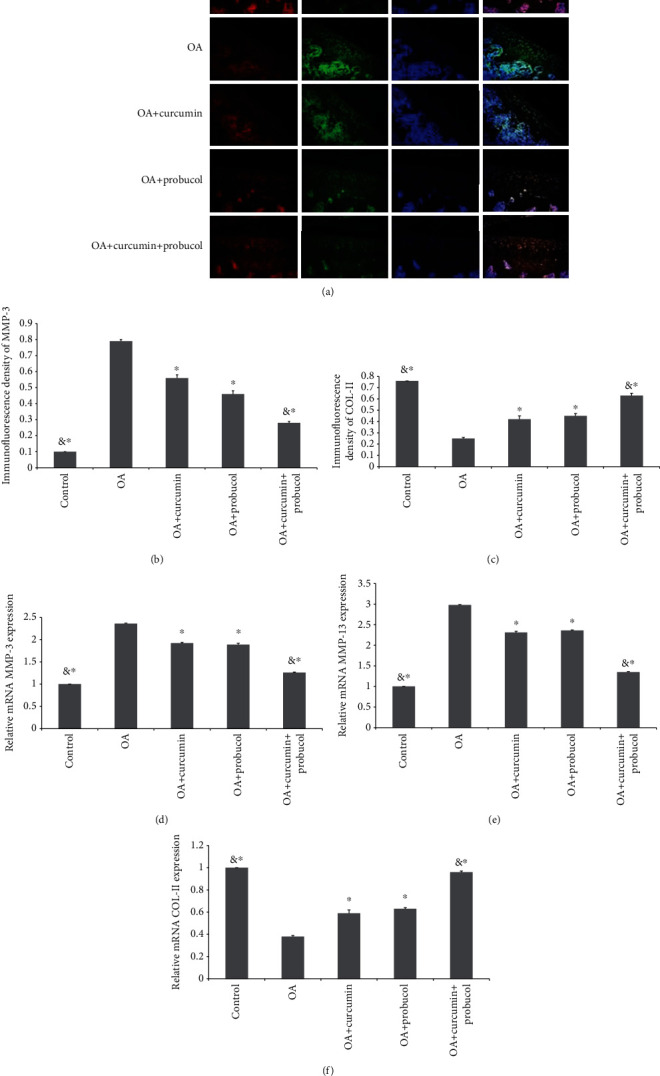
Probucol and curcumin treat OA in vitro and in vivo. (a–c) Quantification results of COL-II and MMP-3 proteins are shown by immunohistochemistry staining (magnification ×200). (d–f) The RT-PCR results for COL-II, MMP-3, and MMP-13. & and ∗ indicate *P* ≤ 0.01 and 0.05 vs. OA, respectively. All experiments were carried out three times.

**Figure 8:**
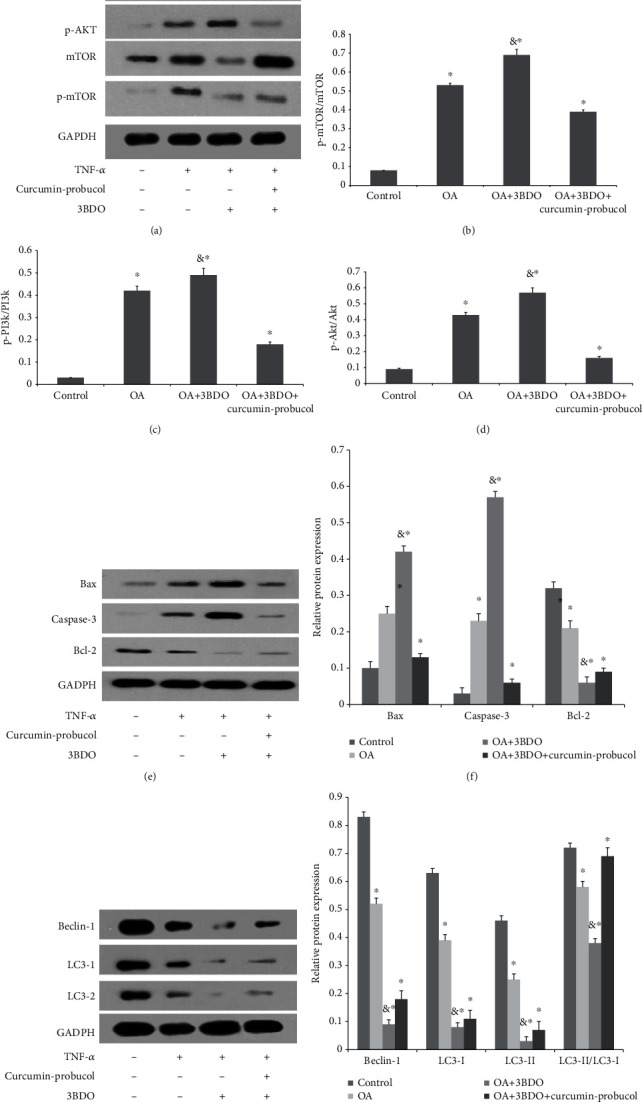
Probucol and curcumin treat OA cartilage by targeting the PI3K-Akt-mTOR pathway. (a–d) Western blot and quantification results of PI3K, p-PI3K, AKT, p-AKT, mTOR, and p-mTOR proteins in rat articular cartilage. (e and f) Quantification results of apoptosis-related proteins (Caspase-3 and Bax) and antidegradation proteins. (g and h) Quantitative results of autophagy-related protein expression (beclin-1 and LC3). & and ∗ indicate *P* ≤ 0.01 and 0.05 vs. control, respectively. All experiments were carried out three times.

Subsequently, the effects of probucol and curcumin on inflammatory factors in experimental animals were investigated. The immunohistochemistry assays and RT-PCR verified the expression of ECM-related genes, namely, COL-II, MMP-13, and MMP-3. RT-PCR confirmed that the expression of MMP-13 and MMP-3 was induced by TNF-*α*, but the expression of COL-II was inhibited. However, both probucol and curcumin could significantly inhibit the degradation of COL-II while suppressing the expression of MMP-3 and MMP-13, and the combined administration of the two chemicals can inhibit the expression of inflammatory genes in OA chondrocytes (Figures 7(a)–7(f)). Moreover, 3BDO was used to verify the therapeutic effects of probucol and curcumin as potential antagonists for mTOR signaling. As is seen in the figures below, 3BDO, an agonist of the PI3K-Akt-mTOR pathway, could enhance the expression of p-PI3K/PI3K, p-AKT/AKT, and p-mTOR/mTOR, but curcumin-probucol acted as antagonists against 3BDO (Figures 8(a)–8(h)).

### 3.7. Effects of Curcumin and Probucol on GAG Release

**Figure 9:**
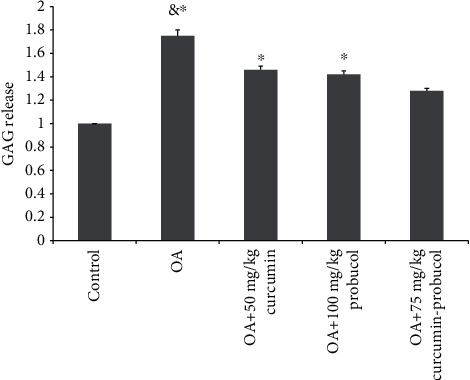
The percentage of cartilage GAG released into the medium at various curcumin and probucol concentrations. & and ∗ indicate *P* ≤ 0.01 and 0.05 vs. control, respectively. All experiments were carried out three times.

Lastly, compared to control, GAG release was reported to show a significant increase in OA chondrocytes. The results from this study suggested that probucol or curcumin could reduce OA-stimulated GAG release when applied alone. However, the combined administration of such two substances via intramuscular injection could significantly (*P* < 0.001) reduce the percentage of OA-stimulated GAG release to a level similar to control (Figure 9). As a result, it is fair to infer that the combined medication of curcumin and probucol could inhibit OA by reducing GAG Release.

## 4. Discussion

One reason why only modest benefit can be obtained from early antiproinflammation cytokine therapy for OA treatment is because of the “one size-fits--fits-all” approach [20, 21]. Actually, from a clinical point of view, OA exhibits great etiological heterogeneity, which was not addressed in those early studies [22]. However, increasing evidence suggests that new OA therapies require a paradigm shift that considers OA as a complex and heterogeneous disease involving interactions among multiple organ systems, not merely a joint disease.

Hypercholesterolemia can lead to severe metabolic diseases; such mellitus characteristics increase the risk of OA [20]. The possible mechanisms behind such phenomenon may be explained as follows [22]: (1) as cholesterol is the primary component of cell membrane, its variations can alter the membrane fluidity and compromise its function. Specifically, the ABCA1 gene, which is involved in cholesterol reverse transport [23], mediates lipid efflux from cells, and changes in this gene may be relevant to OA; (2) hypercholesterolemia may impair mitochondrial function and increase oxidative stress, leading to the progress of OA [24]. Regulation of cholesterol levels in chondrocytes can help maintain normal mitochondrial functions; (3) in hypercholesterolemia, the accumulation of LDL-based lipoproteins in the cartilage ECM also can trigger inflammation. Again, the ABCA1 gene is involved in this process.

The catabolic environment in OA is favored for chondrocyte apoptosis, and it restricts autophagy, which plays a physiologically protective role from harmful stress. The activation of the PI3K/Akt/mTOR pathway not only promotes the expression of P62, an autophagy-related factor, but also affects the expression of apoptosis factors (Bcl-2 and Bax). Besides, this pathway is also involved in the activation of inflammation. Such effects eventually lead to higher MMP-3 and MMP-13 expression in chondrocytes and cartilage. Moreover, the expression of COL-II, a synthetic factor of ECM, is inhibited by the activation of the PI3K-Akt-mTOR pathway, promoting the development of OA [17]. Therefore, inhibiting this pathway is of particular importance for OA therapy [25].

At present, some cholesterol regulatory drugs can inhibit the synthesis of cholesterol, thus, being of potential therapeutic significance for OA [26]. Curcumin and probucol were both reported as cholesterol regulators [27, 28]. Specifically, curcumin can inhibit cholesterol metabolism, while probucol can be used to treat atherosclerosis by inhibiting TNF-*α*-induced cholesterol metabolism [29]. Moreover, the two substances have both been reported to inhibit the PI3K/Akt/mTOR pathway [30, 31], and the PI3K pathway is involved in the regulation of mitochondrial activity and ROS production [32]. Specifically, curcumin can regulate several signal transcription pathways and function primarily by inhibiting the activity of NF-ƙB, a transcription factor associated with inflammation [33]. And probucol can inhibit atherosclerosis in mice by promoting ECM production [34]. In addition, curcumin treats OA by maintaining the levels of ECM-related factors, mainly COL-II and Sox-9 [35]. Based on these previously published findings, the two chemicals were investigated as potential candidates for OA therapy in the present study, and TNF-*α* was selected as the inflammatory stress cytokine of the chondrocytes.

In the present study, reduced autophagy and increased apoptosis in chondrocytes were observed after TNF-ɑ stimulation, which is inconsistent with the results from Shakeri's report [36]. With combined administration of curcumin and probucol, autophagy was promoted and apoptosis was suppressed, indicating their protective effects on chondrocytes. However, aside from proliferation and apoptosis, cell migration also plays an important role in biological processes, and chondrocyte migration is especially necessary for cartilage repair [37, 38]. Previous researches have shown that chondrocytes migrate in vitro in response to chemoattractant factors, such as cytokines, growth factors, and ECM components. The dysfunctional migration of chondrocytes leads to their aggregation in OA and changes in ECM components [39]. However, in this study, curcumin and probucol were verified to promote chondrocyte migration, further suggesting their therapeutic potential for OA.

To explore the signaling pathways involved in curcumin-probucol treatment, further studies with 3BDO, a PI3K/AKT/mTOR signaling pathway agonist, were conducted. The results confirmed that curcumin and probucol antagonize 3BDO and thereby inhibiting the PI3K/AKT/mTOR signaling pathway, alleviating cartilage destruction in OA. Besides, morphological studies on cartilage were also performed to evaluate cartilage degeneration in vivo, and the results further verified the protective effects of such two substances on the cartilage matrix. Moreover, when applied alone, both curcumin and probucol can promote the expression of COL-II in ECM while inhibiting the expression of MMP-3 and MMP-13. A combined administration of such two chemicals would produce synergistic protective effects, and such findings were verified both in vitro and in vivo.

*Curcuma longa*, from which curcumin is extracted, has a history of thousands of years in Asian countries as a dietary spice and a therapeutic agent [40]. However, it was not until the mid-20th century that the biological properties of curcumin were scientifically identified [41]. Recent researches suggested that curcumin might take function in OA therapy [42–46]. Moreover, Henrotin et al. stated that even curcumin has not been considered as a recommended intervention for OA treatments, yet it still should be investigated for its safety and efficacy [22]. Some recent publications verified that different cell types have its specific permeability characteristics towards curcumin intake, and different intracellular curcumin concentrations might control its performance in vivo [23, 24]. In vitro experiments verified that 20 *μ*M curcumin would not bring toxicity to human articular cartilage [45]. However, at a concentration of 50 *μ*M, it will lead to a decrease in the survival rate of chondrocytes [46, 47].In our study, the optimal curcumin concentration to treat OA was determined to be 50 *μ*M, but due to the potential considerable toxicity to normal chondrocytes at such concentration [48], caution still must be taken when applying curcumin for clinical treatment of OA.

Furthermore, lower aggrecan loss in IL-1*β*-stimulated articular cartilage explants has been reported to occur at a curcumin concentration no lower than 100 *μ*M [49]. Additionally, it was reported that probucol would also bring side effects, such as ventricular arrhythmia, torsades de pointes, and syncope [50]. Probucol can induce long-term QT syndrome by blocking the ethera-go-go-related genes [36]. Moreover, the bioavailability of curcumin is also very low because of its rapid metabolism, poor solubility and stability in aqueous solutions, and extensive binding to plasma proteins. A pharmacokinetic study suggested that curcumin is not absorbed adequately from the gastrointestinal tract. The peak serum concentration of curcumin was found to be only 1.77 *μ*M even with the highest dose of 8000 mg/day [51], which means its serum concentration in people who take in only 2 grams of curcumin is nearly undetectable [40]. In this study, the dosages of both chemicals were carefully designed to avoid possible side effects; meanwhile, we intended to verify whether the substances would still bring protective effects on chondrocytes and cartilage at such low concentrations. In this study, curcumin dosages that are close to its actual concentration in the human body were applied even though they are not enough to protect the cartilage in terms of total maintenance of proteoglycans. However, the results still suggested that a combined administration of the two chemicals can enhance the autophagy of chondrocytes, promote their proliferation, inhibit their apoptosis, increase their migration, and maintain their mitochondrial functions and the stability of the cartilage ECM by inhibiting the PI3K/Akt/mTOR pathway, and these findings had been confirmed in our in vivo animal studies.

Cholesterol regulatory drugs have various functions related to immune regulation and cartilage protection [52], and they may be promising candidates to alleviate cartilage degeneration. However, there are still several questions to be resolved, and the most important one is to what extent should cholesterol levels be lowered so that they can be always maintained at the level required for the stability of the internal cartilage environment. Besides, OA patients should be further categorized into different phenotypes based on their cholesterol metabolism situation.

In summary, OA patients are frequently accompanied with metabolic disorders, so it is necessary to regard the pathological mechanisms of OA as a metabolic disorder syndrome. Apart from their effects in regulating cholesterol levels, curcumin and probucol can also maintain chondrocyte stability by balancing cell autophagy and apoptosis. Moreover, lowering cholesterol levels by medication or changing food intake and lifestyle is one of the fundamentals to prevent the onset and progression of OA, and a combined administration of curcumin and probucol is promising for OA prevention and treatment.

## Data Availability

The data used to support the findings of this study are available from the corresponding author upon request.
